# Remarks on the Microscopic Characters of the Urine in Bright’s Disease of the Kidney

**Published:** 1852-07

**Authors:** John D. Macdonald


*Remarks on the Microscopic Characters of the Urine in Bright's Disease of the Kidney.* By JOHN D. MACDONALD, ESQ., R. N.

Having studied, from time to time, the microscopic characters of the urine in Bright's disease of the kidney, and compared the appearances there presented with those of a section, or torn-up portion of the diseased gland itself, when the occasion offered, several objects of interest were frequently to be observed, either little known or not at all described; and, as they illustrate and support the pathological views of the malady in question, so ably put forward by Dr. George Johnson, they may be worthy of notice.

The presence of lymph, or pus, is supposed to afford the only unequivocal evidence of inflammatory action, and this seems to be borne out in renal disease. Mechanical obstruction and passive congestion are the first effects of excessive distension of the uriniferous tubules, by whatever cause induced, and in the great majority of cases the antecedents of any active state which the vessels may take on. The condition of the kidney in these respects will be clearly indicated by the presence or absence of fibrinous casts of the tubules in the urine; but this part of the subject must be dismissed, as the remarks about to be offered chiefly refer to certain changes which are incident to the secreting cells of the kidney.

Admitting the faithfulness of the exposition given by Mr. Bowman, of the minute anatomy of the kidney, as demonstrated at any moment, a vindication of it may be called for, the pathology of the granular kidney seems to be simply this—an error of secretion, depending most probably upon the faulty character of the plasma from which it is derived, is attended with a gradually increasing deposition of oily matter within the cells, which are the principal agents in the process. The circumstance gives rise to an over-distension of the tubules, (more especially between the meshes of the matrix, where there is least resistance,) and thus, by mechanical pressure, the return of blood through the venous plexus which surrounds them is obstructed, the Malpighian tufts become congested, and may be ultimately ruptured; so that the presence of albumen in one instance, and actual blood in the other, may be detected in the urine. In some cases, the hæmorrhage which occurs in this way is truly alarming, and the more immediate cause of a fatal issue. Notwithstanding the close analogies existing between the liver and kidney, with respect to structure, function, and the diseases to which they are subject, there is this much to be said, that the kidney does not admit of congestion with impunity, while the liver may be gorged to a considerable extent, and yet suffer comparatively little disorder of function. There may be often some difficulty in determining the fatty nature of the contents of the epithelial cells of the kidney, when discharged with the urine, more especially if little is understood about the changes which they are liable to undergo, in obedience to common laws. The most usual metamorphoses of these cells exhibit eight phases. In the first, distinct oil globules are to be seen within them, varying in number and size, and giving a more or less rounded figure to the cells themselves, which become enlarged in a proportionate degree. 2dly. The oil globules may run together, and so completely fill the cells, that (with the exception of the nuclei, which are generally visible,) they appear like starch granules, or little masses of oil, refracting the light powerfully. 3dly. The fat gradually assumes a more concrete form, loses much of its brilliancy, and presents a mealy surface, any remaining globules becoming angular and irregular in form. 4thly. This concrete mass accumulates around the nucleus, and a clear space begins to appear between it and the cell-wall on the opposite side, which may be partly due to the imbibition of water. 5thly. An analytical change takes place in the fatty matter, which separates into its two constituents, the more fluid part exuding through the cell wall, while the more solid presents a crescentic appearance, the nucleus occupying its centre. 6thly. The cell itself breaks down at its weakest part, and the crescentic mass exhibits a well defined convex border, formed by the remaining portion of the cell, and an uneven concavity where the cell is defective, and the concrete fat is exposed. 7thly. The little crescent continues to extend itself, the outline becomes better defined, and a fusiform figure is ultimately assumed, the nucleus still occupying the centre of the mass, having undergone no apparent change; and 8thly. The pointed extremities of this little fusiform body are drawn out into filamentous processes, so that when several of these altered epithelial particles unite end to end, as often happens, one is reminded of the mode of development of the white fibrous tissue; and on the whole they present such a close resemblance to cancer cells, that the history of their formation is of some importance, to be borne in mind with reference to diagnosis, especially if blood globules be also present with them; for it has been asserted, that if fusiform cells and blood discs are found to co-exist in the urine, certain indication is afforded of cancerous disease, affecting either the kidney or the bladder.

Now, many persons, keeping this doctrine in view, may arrive at once at a conclusion, without waiting to draw the distinction between an actual cell and a cell-like body. Forms very similar to those above described, abound in the spleen, with blood discs seemingly as nuclei, and differing altogether from the muscular fibre cells (so called) of the tubercular sheaths.—*Lond. Med. Times and Gazette.*

